# Unsupervised Clustering Techniques Identify Movement Strategies in the Countermovement Jump Associated With Musculoskeletal Injury Risk During US Marine Corps Officer Candidates School

**DOI:** 10.3389/fphys.2022.868002

**Published:** 2022-05-11

**Authors:** Matthew B. Bird, Qi Mi, Kristen J. Koltun, Mita Lovalekar, Brian J. Martin, AuraLea Fain, Angelique Bannister, Angelito Vera Cruz, Tim L. A. Doyle, Bradley C. Nindl

**Affiliations:** ^1^ Neuromuscular Research Laboratory/Warrior Human Performance Research Center, Department of Sports Medicine and Nutrition, University of Pittsburgh, Pittsburgh, PA, United States; ^2^ Biomechanics, Physical Performance and Exercise Research Group, Department of Health Sciences, Macquarie University, Sydney, NSW, Australia; ^3^ USMC Officer Candidates School, Quantico, VA, United States

**Keywords:** musculoskeletal injuries, markerless motion capture, force plates, marines, screening, unsupervised learning, k-means clustering, military

## Abstract

Musculoskeletal injuries (MSKI) are a significant burden on the military healthcare system. Movement strategies, genetics, and fitness level have been identified as potential contributors to MSKI risk. Screening measures associated with MSKI risk are emerging, including novel technologies, such as markerless motion capture (mMoCap) and force plates (FP) and allow for field expedient measures in dynamic military settings. The aim of the current study was to evaluate movement strategies (i.e., describe variables) of the countermovement jump (CMJ) in Marine officer candidates (MOCs) *via* mMoCap and FP technology by clustering variables to create distinct movement strategies associated with MSKI sustained during Officer Candidates School (OCS). 728 MOCs were tested and 668 MOCs (Male MOCs = 547, Female MOCs = 121) were used for analysis. MOCs performed 3 maximal CMJs in a mMoCap space with FP embedded into the system. De-identified MSKI data was acquired from internal OCS reports for those who presented to the OCS Physical Therapy department for MSKI treatment during the 10 weeks of OCS training. Three distinct clusters were formed with variables relating to CMJ kinetics and kinematics from the mMoCap and FPs. Proportions of MOCs with a lower extremity and torso MSKI across clusters were significantly different (*p* < 0.001), with the high-risk cluster having the highest proportions (30.5%), followed by moderate-risk cluster (22.5%) and low-risk cluster (13.8%). Kinetics, including braking rate of force development (BRFD), braking net impulse and propulsive net impulse, were higher in low-risk cluster compared to the high-risk cluster (*p* < 0.001). Lesser degrees of flexion and shorter CMJ phase durations (braking phase and propulsive phase) were observed in low-risk cluster compared to both moderate-risk and high-risk clusters. Male MOCs were distributed equally across clusters while female MOCs were primarily distributed in the high-risk cluster. Movement strategies (i.e., clusters), as quantified by mMoCap and FPs, were successfully described with MOCs MSKI risk proportions between clusters. These results provide actionable thresholds of key performance indicators for practitioners to use for screening measures in classifying greater MSKI risk. These tools may add value in creating modifiable strength and conditioning training programs before or during military training.

## Introduction

Musculoskeletal injuries (MSKIs) sustained during initial military training remain a significant cause of lost duty time, attrition, and financial burden on the military healthcare system, as well as degrade military readiness and subsequent deployability (Piantanida et al., 2000; Nindl et al., 2013a; Nindl et al., 2013b; Lovalekar et al., 2021). According to the Army Public Health Command's, Health of the Force Report 2020, over 50% of soldiers experienced an injury resulting in 2 million medical encounters and 10 million limited duty days (APHC, 2020). Consequently, there is heightened awareness and interest in screening tests that can inform military leadership regarding MSKI risk and be incorporated into policy and practices to mitigate training related MSKIs.

MSKIs sustained during military training are multifaceted and can be attributed to a host of factors, such as genotype (Bray et al., 2009), low fitness (Robinson et al., 2016), female sex (Lovalekar et al., 2020), or prior MSKI history (Eagle et al., 2019). Additionally, movement strategies have been associated with MSKI risk in both athletic and military populations (Chorba et al., 2010; Lisman et al., 2013; Markström et al., 2019). Previous attempts to evaluate movement strategies have included the gold-standard method of marker-based motion capture (MoCap) (Russell et al., 2006). Although the data is clinically meaningful, MoCap is burdensome on time and largely confined to state-of-the-art biomechanical laboratories and thereby preventing field data collection. In an attempt to move “from the lab to the field” and to address limitations of MoCap, emerging technologies and algorithms have been developed as alternative testing modalities. Markerless motion capture (mMoCap) is an emerging technology (Mündermann et al., 2006; Sonnenfeld et al., 2021) for movement screening that reportedly produces valid ground reaction force estimates (Fry et al., 2016; Mosier et al., 2019), valid to MoCap system kinematics (Perrott et al., 2017; Drazan et al., 2021), and is reliable (Martinez et al., 2018; Mosier et al., 2018). mMoCap may provide a field expedient measure to evaluate movement strategy-related kinetics, kinematics and performance measures and provide insight into MSKI risk. In addition, the incorporation of concurrent FP and mMoCap assessment measures could create a higher fidelity system to detect kinetics in different phases of commonly performed dynamic movements, such as the countermovement jump (CMJ) (Beckham et al., 2014; McMahon et al., 2018).

One particular movement, the CMJ, may be beneficial for incorporation with FP and mMoCap testing as it has been used widely as a screening and readiness measure for athletes and military personnel as it is directly correlated to isometric strength, one repetition maximum half squat (Boraczyński et al., 2020), and is reliable and repeatable for quantifying neuromuscular readiness (Cormack et al., 2008; Welsh et al., 2008; Merrigan et al., 2020). The CMJ is a simple and field-ready test that may provide information regarding MSKI risk and preventative strategies to mitigate MSKIs through information, such as force production, loading kinematics *via* FP or mMoCap technology (Hart et al., 2019; Pontillo and Sennett, 2019; Pontillo et al., 2021).

To analyze movement strategies associated with MSKI risk, independent statistics (i.e., T-tests, ANOVAs) and univariate prediction modeling (i.e., logistic and linear regression) have traditionally been used indicate and predict MSKI (Dudley et al., 2017; de la Motte et al., 2019), whereas more robust analytical, statistical, and machine learning approaches have been underutilized for MSKI prediction. These advanced methodologies may better indentify the relevant information and complex relationships associated with MSKI and provide an appropriately robust approach to a non-linear problem (i.e., indicating MSKI risk), but have yet to be fully evaluated in this manner. Specifically, supervised learning that utilizes labeled data such as MSKI or noMSKI to train an algorithm for prediction (Connaboy et al., 2018) and unsupervised learning, which uses unlabeled data to detect trends or hidden patterns within the data set (i.e., Clustering), warrant further investigation for identifying MSKI risk.

To date, cluster analysis, a specific type of unsupervised learning, has been incorporated into human performance investigations to evaluate physical performance standards (Allison et al., 2019; Gaudet et al., 2019), questionnaires (i.e., pre-game expectations) (Kumar et al., 2019; Popovych et al., 2020), shoulder injuries in volleyball players (Gaudet et al., 2019), and change of direction movement strategies during ACL injuries (Sigurðsson and Briem, 2019). More recently, Rauch et al. (2020), used k-means clustering, a type of clustering method to describe kinetic and kinematic variables that accurately describe CMJ movement strategies for basketball players positional groups. However, clustering has not been applied to assess MSKI risk *via* movement strategies during military training. Therefore, we explored movement strategies associated with MSKI by clustering CMJ kinetic and kinematic variables in FP and mMoCap in Marine Corps Officer candidates (MOCs) undergoing 10 weeks of arduous military training known to cause a high incidence rate for MSKIs (Cumulative injury incidence in Marine Officer Candidates School: *Male MOCs* = 59.5%, *Female MOCs* = 80% (Piantanida et al., 2000). The primary aim of the current study was to evaluate movement strategies (i.e., description of variables) of the CMJ in MOCs *via* mMoCap and FP technology by clustering variables to create distinct movement strategies associated with MSKI sustained during Officer Candidates School (OCS). In addition, we explore other analytical techniques such as two-way ANOVA and provide rationale why movement strategies may be better suited for clustering techniques to assess MSKI risk.

## Materials and Methods

Researchers briefed and consented MOCs for the study. Ethical approval was provided by The University of Pittsburgh (STUDY19030386) and the research was endorsed by the Office of Naval Research and Officer Candidates School. 728 MOCs (Male MOCs = 599, Female MOCs = 129) volunteered and participated in the mMoCap and FP testing which comprised four intake classes.

### Officer Candidates School

One pathway to be commissioned as an Officer in the United States Marine Corps (USMC) requires the completion of OCS. OCS is a 10-week initial military training course for male and female MOCs that consists of intense physical and military training within a controlled and challenging environment. Physical training is conducted based on a predetermined schedule and includes graded events that are designed to test general strength and endurance under field and tactical conditions. In addition, running, hiking, obstacle course navigation and bodyweight exercises are performed as part of regular supervised training occurring 3–5 days/week.

### Movement Assessment

Prior to the start of physical training, height and weight were recorded by a stadiometer and digital scale (Healthometer Professional 500KL, McCook, IL). MOCs were required to perform a warm-up and familiarization phase before testing. DARI mMoCap (DARI Motion, Inc. Overland Park, KS), a 3-dimensional mMoCap system was used for data collection. 8 Black-fly FLIR GigE cameras (50 Hz) were placed around an 2.5 × 3.5 m matted area with Hawkin Dynamic dual FP (Hawkin Dynamics, ME), sampling at 1,000 Hz, embedded into the mat. Prior to daily testing, the DARI mMoCap was calibrated to the manufacturer’s specifications, and FPs were tested to ensure device ground contact. DARI mMoCap use Captury Live motion tracking software (CL, The Captury Ltd., Saarbrücken, Germany) that uses sums of spatial Gaussian functions to generate a subject-specific body model representing the shape and color statistics to estimate joint centers (Stoll et al., 2011). Before capture, the FPs were zeroed and a background subtraction was performed on the DARI mMoCap system so that when MOCs enters the mMoCap area, MOCs are differentiated from the background during initialization of the tracking model. MOCs placed one foot on each FP, and cued into a calibration position, in which both elbows were at 90°, and hands downwards. A computerized subject-based model was generated and virtually overlaid on the live image of the MOCs, and scaling actions (lunges, squats, arm rotations) were performed to capture the MOCs joint centers.

MOCs performed three maximal-effort CMJs, with 15 s rest between each jump. The MOCs were cued to start with hands above head, stand still (1 s of quiet phase to register system weight), and performed the jump with a counter-movement and arm swing to a self-selected depth. Participants were instructed to jump immediately after researchers verbally gave a 3-2-1 countdown. A trial was unsuccessful and redone if the MOC failed to land within the confines of the force plates. If the skeleton was visually misaligned from a joint center, either the MOCs would redo the CMJ or the skeleton would be re-tracked post-hoc. MOCs flagged for a misalignment in the skeleton were further evaluated visually for misalignments in skeleton joint centers. After visual inspection, the mean for each FP and mMoCap variable were calculated and three standard deviations above or below the mean were visually inspected for the potential removal of trail (i.e., mMoCap: Skeleton misalignment or joint center deviating from joint center and FP: Unweighting phase starting early due to movement artifact from MOCs). A total of 728 MOCs were tested, while 668 MOCs (Male MOCs = 547, Female MOCs = 121) were retained after filtering of artifacts in mMoCap and FPs.

**FIGURE 1:**
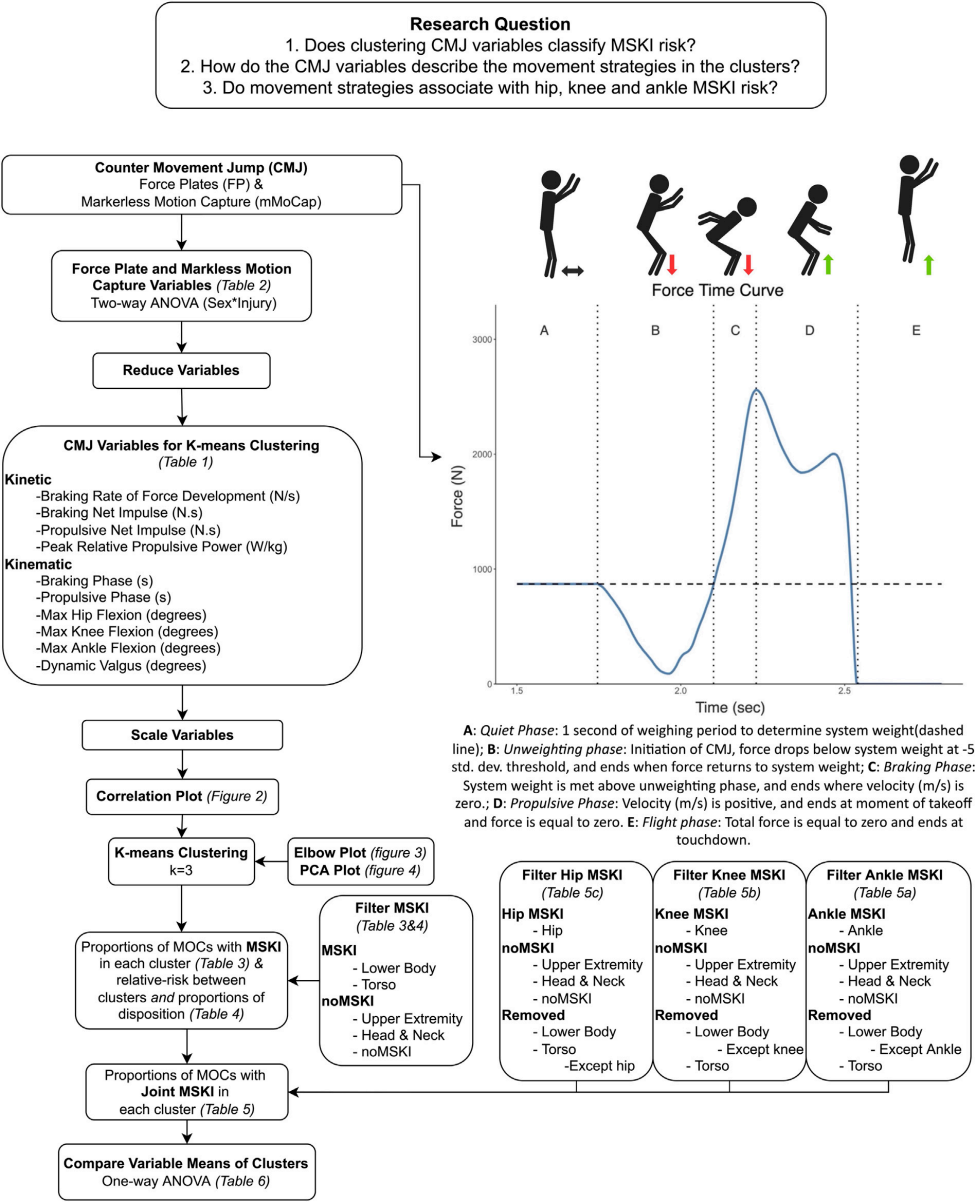
Analytical pipeline describing the data analysis methods, and the force plate force-time curve describing the different phases of the CMJ.

All variables from DARI mMoCap were uploaded to DARI’s cloud platform and processed using DARI Insight Processing (version 1.0.4-250) and DARI Insight Vault (version 1.0.3-854) software. mMoCap joint coordinate systems are defined during movement tracking and calculations for knee, hip and ankle kinematics follow the methods prescribed by the International Society of Biomechanics (Grood and Suntay, 1983; Wu et al., 2005). Variables from FPs were uploaded to Hawkin Dynamic Cloud (v16.2.0) and processed using Hawkin Dynamic Software (v7.3.8) using force-time curve cut-offs for varying phases (i.e., Braking and Propulsive phase) explained in Figure 1, along with the description of variable calculations in Table 1. Analysis was performed on one CMJ that was based on peak CMJ jump height *via* FPs with the matching jump number from mMoCap. DARI mMoCap outputs right and left limbs, which limbs were averaged for a gross bilateral movement (i.e., (Left + Right Ankle Flexion)/2).

**TABLE 1:** K-means clustering variables definitions.

Variables	Definitions
**Kinetic measures**	
Braking RFD (N/s)	Difference in newtons from the end of braking to the start of braking divided by the duration of the braking phase
Braking net impulse (N.s)	Impulse above system weight during braking phase
Propulsive net impulse (N.s)	Impulse above system weight during propulsive phase
Peak relative propulsive power (W/kg)	Peak power during propulsive phase divided by kg of subject mass
**Kinematic measures**	
Braking phase (s)	Duration of braking phase
Propulsive phase (s)	Duration of propulsive phase
Max hip flexion (degrees)^a^	Max flexion angle between pelvis and femur during loading phase
Max knee flexion (degrees)^a^	Max flexion angle between femur and tibia during loading phase
Max ankle flexion (degrees)^a^	Max flexion angle between tibia and foot during loading phase
Dynamic valgus (degrees)^a^	Measure of knee deviation from the leg Plane, which is defined using the positions of hip and ankle joint centers and the pelvis anterior direction

a Markerless Motion Capture variables are from the start of the movement to the pelvis reaching minimum height; Force plate variables are described in Figure 1.

### Musculoskeletal Injuries Labeling

De-identified MSKI data was acquired from internal OCS reports for those who presented to the OCS Physical Therapy department for MSKI treatment during the 10 weeks of OCS training. MSKIs were presented into four anatomical regions: 1) lower body (LB), 2) torso, 3) upper body (UB), 4) head and neck (HN). The specific body parts associated with each region include: 1) *LB*: foot, ankle, knee, lower leg, and upper leg, 2) *Torso*: lumbar spine, thoracic spine, ribs, hip 3) *UB:* shoulder, elbow, upper arm, forearm, hand, and wrist 4) *HN*: cervical spine. Severity of injury was defined as Disposition and was either *Light Duty:* limitations to full training or *Full Duty:* No limitations to training. To account for inclusion of multiple MSKIs within a MOC, injury severity was rounded up to the most severe (i.e., light duty) when analyzing for light duty MSKIs per cluster, and joint MSKI was rounded to the specific joint MSKI analyzed (i.e., Hip MSKI, Knee MSKI, Ankle MSKI). The rounding technique was performed so that the correct joint MSKI and the highest severity was pulled, and so that no duplicate MOCs were presented in the data.

### Data Analysis

Two-way independent measures analysis of variance (ANOVAs) were conducted to analyze the effect of sex (between-subjects variable: male and female), the effect of injury (between-subject variable: MSKI and noMSKI), and the effect of interaction between sex and injury, on 18 FP and mMoCap dependent variables in Table 2. If the interaction effect was statistically significant, simple main effects of injury at each level of sex were analyzed.

**TABLE 2:** Force plate and markerless motion capture variables in Marine Officer Candidates (MOCs).

		Male MOCs (*n* = 547)			Female MOCs (*n* = 121)		
	Abbreviation	MSKI (*n* = 109)	noMSKI (*n* = 438)	Male MOCs (*n* = 547)	MSKI (*n* = 44)	noMSKI (*n* = 77)	Female MOCs (*n* = 121)
**Kinetic variables**							
Braking RFD (N/s)	BRFD	3,349 ± 1,675	3,782 ± 1,832	3,696 ± 1,808	2,180 ± 1,153	2,524 ± 1,223	2,399 ± 1,205
Avg. braking force (N)	ABF	1,190 ± 207	1,235 ± 201	1,226 ± 203	907 ± 128	963 ± 147	943 ± 143
Avg. relative braking force (%BW)	ARBF	152.7 ± 19.6	156.4 ± 19.1	155.6 ± 19.3	147.7 ± 18.5	150.2 ± 16.6	149.3 ± 17.3
Avg. propulsive force (N)	APF	1,364 ± 198	1,404 ± 208	1,396 ± 206	995 ± 125	1,044 ± 144	1,026 ± 139
Avg. relative propulsive force (%BW)	ARPF	174.9 ± 13.0	177.6 ± 16.3	177.1 ± 15.7	161.6 ± 12.1	162.6 ± 11.9	162.2 ± 12.0
Braking net impulse (N.s)	BNI	109 ± 21	112 ± 21	111 ± 21	76 ± 14	83 ± 15	81 ± 15
Propulsive net impulse (N.s)	PNI	222 ± 33	227 ± 33	226 ± 33	147 ± 21	157 ± 22	153 ± 22
Peak relative propulsive power (W/kg)	PRPP	53 ± 7	54 ± 8	54 ± 8	43 ± 6	44 ± 6	43 ± 6
Peak propulsive power (W)	PPP	4,215 ± 789	4,335 ± 827	4,311 ± 820	2,699 ± 491	2,853 ± 492	2,797 ± 495
**Kinematic variables**							
Braking phase (s)	BP	0.29 ± 0.08	0.27 ± 0.07	0.28 ± 0.07	0.28 ± 0.07	0.28 ± 0.06	0.28 ± 0.06
Propulsive phase (s)	PP	0.39 ± 0.05	0.38 ± 0.06	0.38 ± 0.05	0.40 ± 0.05	0.40 ± 0.06	0.40 ± 0.06
Time to take off (s)	TTTO	1.11 ± 0.14	1.09 ± 0.14	1.10 ± 0.14	1.10 ± 0.14	1.10 ± 0.12	1.10 ± 0.13
Max hip flexion (degrees)^a^	HF	99.9 ± 15.4	97.6 ± 16.1	98.0 ± 16.0	103.0 ± 17.7	105.7 ± 15.5	104.8 ± 16.4
Max knee flexion (degrees)^a^	KF	116.3 ± 14.0	114.7 ± 14.7	115.0 ± 14.6	109.1 ± 11.2	113.3 ± 14.7	111.8 ± 13.6
Max ankle flexion (degrees)^a^	AF	33.7 ± 6.3	32.8 ± 6.0	33.0 ± 6.0	34.5 ± 6.7	33.7 ± 5.1	34.0 ± 5.7
Dynamic valgus (degrees)^a^	DV	5.7 ± 3.4	5.5 ± 3.0	5.5 ± 3.0	6.7 ± 3.4	7.1 ± 3.8	6.9 ± 3.6
**Performance variables**							
Jump height (m)	JH	0.40 ± 0.07	0.40 ± 0.07	0.40 ± 0.07	0.28 ± 0.05	0.29 ± 0.05	0.29 ± 0.05
Modified reactive strength index (JH/TTTO)	mRSI	0.36 ± 0.08	0.38 ± 0.09	0.38 ± 0.09	0.26 ± 0.06	0.27 ± 0.07	0.27 ± 0.07

a Markerless motion capture variables; MSKI = Lower Extremity and Torso; noMSKI = Upper Extremity, Head and Neck, and noMSKI; Two-way ANOVA for Sex*Injury results in text; MSKI = Musculoskeletal Injury; data presented as mean ± standard deviation.

K-means clustering (MacQueen, 1967) is an unsupervised technique that initially randomly assigns cluster centroids, then in an iterative process attempts to group the values closest to the cluster centroid in which to minimize the sum of squares within each cluster. Before k-means clustering, ten variables were reduced and scaled from Table 2 to reduce dimensionality (Steinbach et al., 2004), characterizing the kinetic and kinematic braking and propulsive phases. Each variable was scaled so that its mean equals zero and the standard deviation equals one. Spearman correlations were performed on the ten variables to assess for highly correlated variables that display similar concepts, thus the concepts may be represented twice (Sambandam, 2003). Thresholds of highly correlated variables were set at *r* >0.85 or <−0.85, and if pairwise variables fell >0.85 or <−0.85 one of the pairwise variables would be subject to be removed. To determine the number of clusters, an elbow plot and visual inspection of three separate runs (two, three, and four number of clusters) of k-means were performed. The elbow method is a subjective measure, evaluating for a “kink” in the curve, in which optimal number of clusters are chosen (Figure 3), by measuring the total within sum of squares (Kodinariya and Makwana, 2013). The separate runs of k-means were visualized by a 2-dimensional principal component analysis (PCA) plot (Figure 4) to evaluate for the minimization of cluster overlap. R Version 3.6.1 (R Core Team, 2019) packages including Hmisc V. 4.5 (rcorr, Spearman correlation), stat V. 3.6.2 (kmeans, K-Means Clustering), ggplot2 V. 3.3.5 (Elbow plot and Cluster visualization) were used for analysis and visualization.

Fisher’s exact tests were used to compare the proportions across clusters for MOCs with MSKIs, Male and Female MOCs with MSKI, Female and Male MOCs, MOCs with ankle MSKI, Knee MSKI, and Hip MSKI, Male and Female MOCS with ankle MSKI, Knee MSKI, and Hip MSKI, MOCs with Light Duty MSKI, and Male and Female MOCs with Light Duty MSKI. Relative risk and 95% confidence intervals were calculated between each cluster for each of the proportions. One-way independent measures analysis of variance (ANOVAs) were conducted to analyze the effect of cluster (between-subjects variable: C1,C2,C3), on each of the ten dependent variables used in k-means clustering. All Statistical analysis was conducted using IBM SPSS Statistics Version 25 (IBM Corp; Armonk, NY). Statistical significance was set *a priori* at *α* = 0.05, two-sided.

## Results

Female MOCs were significantly shorter (M = 176.5 ± 6.9 cm; F = 163.9 ± 5.4 cm, *p* < 0.001) and weighed less than male MOCs (M = 80.2 ± 9.4 kg; F = 64.6 ± 7.2 kg, *p* < 0.001), while age was not different (M = 24.8 ± 3.0 years; F = 24.8 ± 3.3 years). Two-way ANOVAs were conducted to analyze the effect of sex and injury on the variables listed in Table 2. There was no significant interaction between Sex and Injury in their effect on any of the dependent variables analyzed (all interaction *p* values >0.05). There was a statistically significant main effect of sex on all dependent variables analyzed (all main effect of sex *p* values < 0.05), except for AF (*p* = 0.183, η_p_^2^ = 0.003), TTTO (*p* = 0.894, η_p_^2^ = 0.000), and BP (*p* = 0.674, η_p_^2^ = 0.000). There were significant main effects for injury for BRFD (*p* = 0.037, η_p_^2^ = 0.007), ABF (*p* = 0.016, ηp2 = 0.009), APF (*p* = 0.037, η_p_^2^ = 0.007), BNI (*p* = 0.029, η_p_^2^ = 0.007), and PNI (*p* = 0.042, η_p_^2^ = 0.006). The main effects of injury were not statistically significantly different for the other dependent variables.

### K-Means Clustering

**FIGURE 2:**
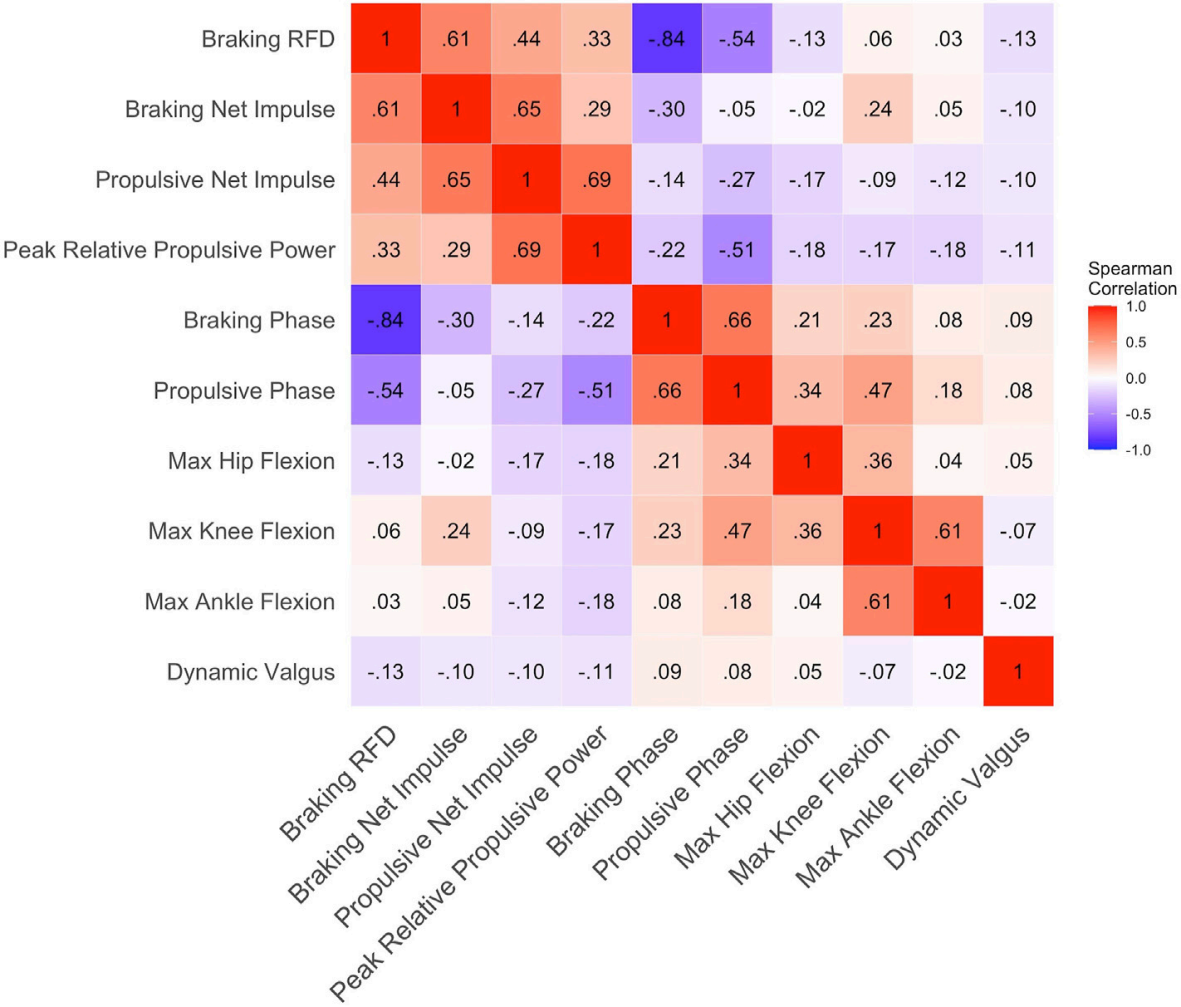
Correlation plot on the 10 variables used for k-means clustering; *r* values represented by value and color representation (darker red = greater positive *r* values and darker blue = greater negative *r* values); If pairwise *r* values were >0.85 and <−0.85 then one variable of the pairwise variables were removed; No variables were removed.

Ten variables were reduced and scaled from Table 2 to characterize the CMJ kinetics and kinematic braking and propulsive phases for k-means clustering. Before clustering, Spearman correlations were performed to check for highly correlated variables and none of the ten variables used for k-means clustering presented with the criterion described in Figure 2 (*r* >0.85 or <−0.85). BP and BRFD had the greatest correlation (*r* = −0.84), while all other pairwise correlations were below *r* = 0.69 and greater than *r* = −0.54.

**FIGURE 3:**
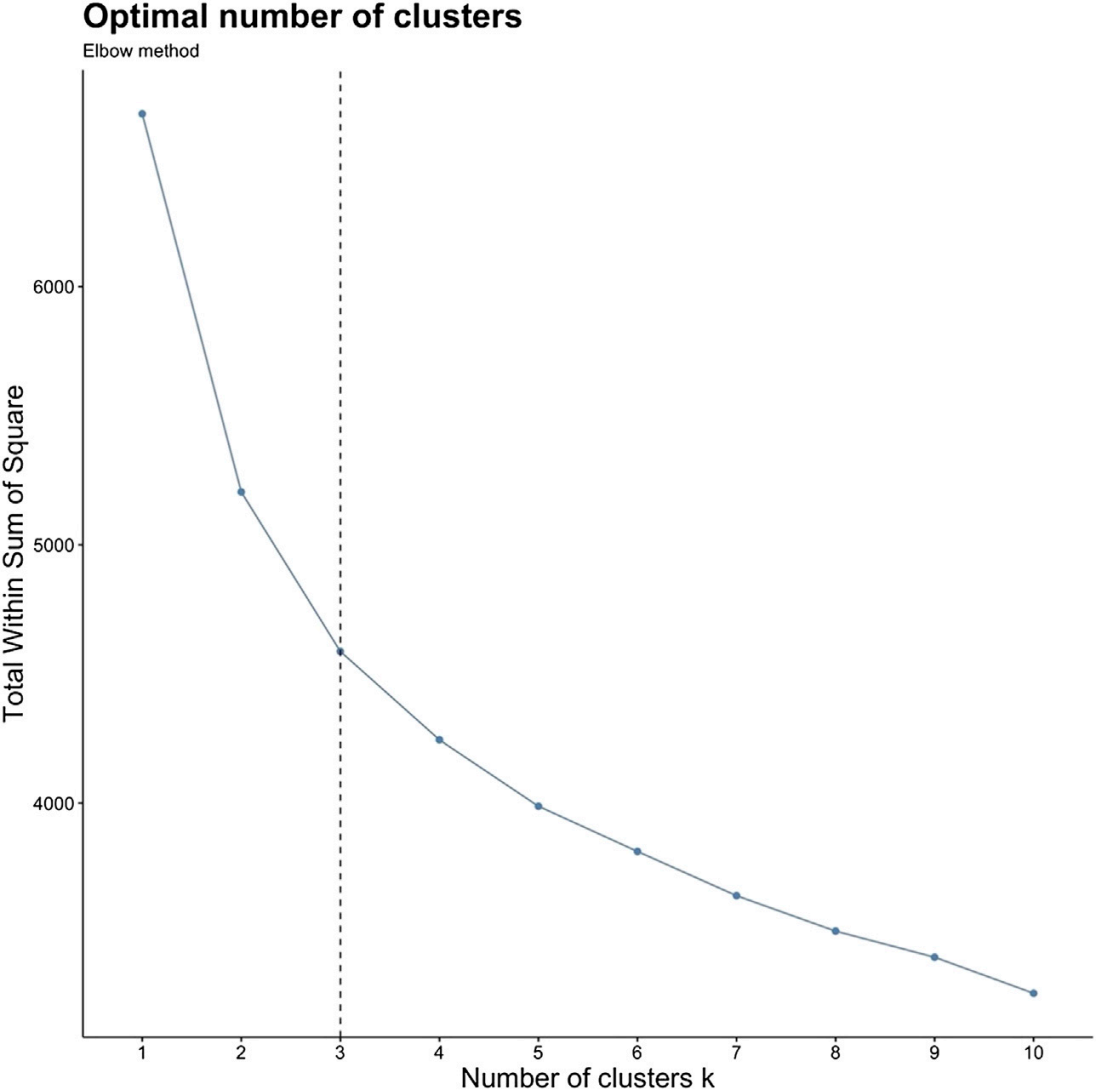
Elbow plot demonstrating, y-axis = total within sum of squares by x-axis = number of clusters (k); Subjective evaluation for number of clusters chosen for analysis, used for determination of k; “Kink” in curve occurs at k = 2 and 3.

**FIGURE 4:**
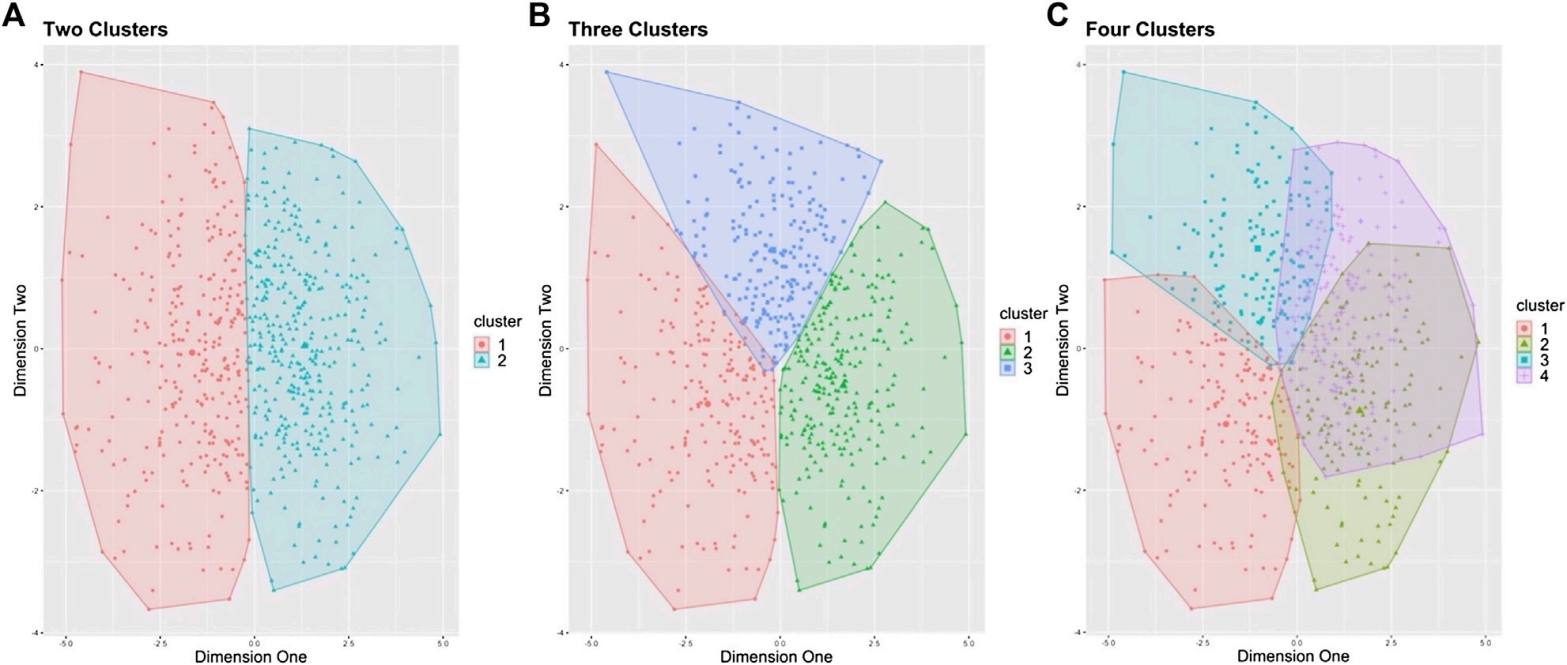
Three separate analyses of k-means clustering (k = 2, 3, and 4). **(A)** k = 2, *Two Clusters*. **(B)** k = 3, *Three Clusters*. **(C)** k = 4, *Four Clusters*; Represented by PCA 2-d plot for representation of cluster distribution and overlap; **(B)** was chosen with insight from elbow plot, and visual comparison of cluster distributions with minimal overlap of clusters.

The ten scaled variables were analyzed using k-means clustering for male and female MOCs (MOCs = 668, Male MOCs = 547, Female MOCs = 121). The Elbow plot determined (Figure 3) the optimal number of clusters were two and three clusters. Clusters were visualized (Figure 4) in a 2-dimensional PCA plot, displaying two and three clusters had the least amount of overlap when compared to four clusters, thus three clusters where chosen.

Fishers exact test was used to compare proportion of MOCs with MSKIs between clusters (Table 3) and showed that clusters were significantly different (*p* < 0.001). C3 having the highest proportion of MSKIs (30.5%) and C1 having the lowest (13.8%), while C2 moderate (22.5%). This defines C3 as a “high-risk” cluster, C1 as a “low-risk” cluster, and C2 as a “moderate-risk” cluster based on proportions MOCs with MSKIs in each cluster. Relative-risk (95% Confidence Interval) (Table 3) compared high-risk to low-risk (RR (95% CI) =2.2 (1.5–3.3)), moderate risk to low risk (RR (95% CI) = 1.6 (1.1–2.5)), and high-risk to moderate-risk (RR (95% CI) = 1.3 (1.0–1.9)). After stratifying by sex MSKI, the percent of male MOCs with MSKI was proportionally different between clusters (*p* = 0.011). While the percent of female MOCs with MSKI were proportionally similar for the low-risk and moderate-risk cluster (25% MSKI), while high-risk (38.1% MSKI) was different, although not significant across clusters (*p* = 0.770). Lastly, proportions of sex were calculated and the percent of female MOCs were primarily distributed to high-risk cluster (41%), while similar distributions in low-risk (3.9%) and moderate-risk (3.8%).

**TABLE 3:** Proportions of MOCs with Lower Body and Torso Musculoskeletal Injury by Cluster.

	C1 (low-risk)	C2 (moderate-risk)	C3 (high-risk)	Fisher’s exact test *p* value	Relative-risk (95% CI)		
					C3/C1	C2/C1	C3/C2
%MSKI	28/203 = 13.8%	47/209 = 22.5%	78/256 = 30.5%	<0.001	2.2 (1.5–3.3)	1.6 (1.1–2.5)	1.3 (1.0–1.9)
%MSKI Male	26/195 = 13.3%	45/201 = 22.4%	38/151 = 25.2%	0.011	1.9 (1.2–3.0)	1.7 (1.1–2.6)	1.1 (0.77–1.6)
%MSKI Female	2/8 = 25.0%	2/8 = 25.0%	40/105 = 38.1%	0.770	1.5 (0.5–5.1)	1.0 (0.2–5.5)	1.5 (0.5–5.1)
%Male	195/203 = 96.1%	201/209 = 96.2%	151/256 = 59.0%	<0.001			
%Female	8/203 = 3.9%	8/209 = 3.8%	105/256 = 41.0%	<0.001			

Fisher's exact test comparing clusters; *p* < 0.05 across all three clusters; Relative-Risk (95% confidence interval) comparing between clusters; MSKI = Musculoskeletal Injury; Each MOCs received a MSKI, or noMSKI label: MSKI = lower body and torso and noMSKI = upper body, head and neck, and noMSKI; **%MSKI** = (MSKI/(MSKI + noMSKI)); **%MSKI Male**=(Male MSKI/(Male MSKI+Male noMKSI)); **%MSKI Female**=(Female MSKI/(Female MSKI+Female noMSKI)); **%Female**=(Female/(Female+Male)); **%Male** = (Male/(Female+Male)).

Disposition of MSKI was assessed by evaluating proportion of MOCs with a light duty MSKI within each cluster (Table 4). There were no significant differences across clusters (*p* = 0.149), with high-risk having largest percentage of light duty MSKI (79.4%) and moderate risk the lowest (64.8%). Joint specific MSKI (Table 5) hip (Table 5C) and knee (Table 5B) MSKI across clusters were not significantly different, while ankle (Table 5A) MSKI was significantly different (*p* = 0.010, low-risk: 3.3%, moderate-risk: 3.6%, high-risk: 10.1%).

**TABLE 4:** Proportion of MOCs with Light Duty Musculoskeletal Injury by Cluster.

	C1 (low-risk)	C2 (moderate-risk)	C3 (high-risk)	Fisher’s exact test *p* value
%MSKI Light duty	21/28 = 75.0%	30/47 = 64.8%	62/78 = 79.4%	0.149
%MSKI Light duty male	20/26 = 76.9%	28/45 = 62.2%	26/38 = 68.4%	0.458
%MSKI Light duty female	1/2 = 50.0%	2/2 = 100%	36/40 = 90.0%	0.394

Fisher's exact test comparing clusters; *p* < 0.05 across all three clusters; Light Duty = Missed training days due to MSKI; MSKI = Musculoskeletal Injury; **%MSKI Light duty**=(Light duty/(Light duty+Full duty)); **%MSKI Light duty male**=(Male light duty/(Male light duty+Male full duty)); **%MSKI Light duty female**=(Female light duty/(Female light duty+Female full duty)).

**TABLE 5:** Proportions of MOCs with Joint Musculoskeletal Injury by Cluster.

	C1 (low-risk)	C2 (moderate-risk)	C3 (high-risk)	Fisher’s exact test *p* value
**A. Ankle MSKI (*n* = 547)**				
%MSKI Ankle	6/181 = 3.3%	6/168 = 3.6%	20/198 = 10.1%	0.010
%MSKI Ankle male	5/174 = 2.9%	6/162 = 3.7%	9/122 = 7.4%	0.166
%MSKI Ankle female	1/7 = 14.3%	0/6 = 0.0%	11/76 = 14.4%	1.00
**B. Knee MSKI (*n* = 557)**				
%MSKI Knee	8/183 = 4.4%	18/180 = 10.0%	16/194 = 8.2%	0.106
%MSKI Knee male	8/177 = 4.5%	17/173 = 9.8%	8/121 = 6.6%	0.153
%MSKI Knee female	0/6 = 0.0%	1/7 = 14.3%	8/73 = 11.0%	0.789
**C. Hip MSKI (*n* = 532)**				
%MSKI Hip	2/177 = 1.1%	5/167 = 3.0%	10/188 = 5.3%	0.072
%MSKI Hip male	2/171 = 1.1%	5/161 = 3.1%	4/117 = 3.4%	0.346
%MSKI Hip female	0/6 = 0.0%	0/6 = 0.0%	6/71 = 8.5%	1.00

Fisher's exact test comparing clusters; *p* < 0.05 across all three clusters; **5a. MSKI Ankle** (*n* = 547): MSKI = Ankle, noMSKI = upper body, head and neck and noMSKI, NA = lower body, and torso MSKIs excluding ankle; **5b. Knee MSKI** (*n* = 557): MSKI = Knee, noMSKI = upper body, head and neck and noMSKI, NA = lower body and torso MSKIs excluding knee; **5c. Hip MSKI** (*n* = 532): MSKI = Hip, noMSKI = upper body, head and neck and noMSKI, NA = lower body and torso MSKIs excluding hip; If labeled “NA” then excluded from analysis; **%MSKI Joint**=(MSKI Joint/(MSKI Joint+noMSKI)); **%MSKI Joint male**=(MSKI Joint male/(MSKI Joint male+noMSKI male)); **%MSKI Joint female**=(MSKI Joint female/(MSKI Joint female+noMSKI female)).

**FIGURE 5:**
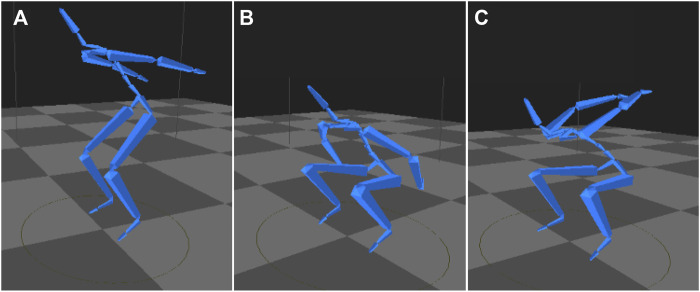
Example of Marine Officer Candidates from each cluster during max joint flexion in the CMJ **(A)** (Left): Low-risk cluster **(B)** (Middle): Moderate-risk cluster **(C)** (Right): High-risk cluster; Cluster names defined by proportions of MOCs with MSKI (Table 3); *Low-risk cluster*: lower joint flexions, shorter time durations, and higher kinetics; *Moderate-risk cluster*: higher joint flexions, moderate time durations, and moderate kinetics; *High-risk cluster:* moderate joint flexions, longer time durations, and lower kinetics.

One-way ANOVAs were conducted on each dependent variable (Table 6), demonstrating that k-means clustering performed well at separating groups for each of the ten variables (*p* < 0.001). Bonferroni Post Hoc identified significant differences in BNI, PNI, PRPP, BP, PP, KF, and AF across the clusters, and BRFD, HF, and DV were significantly different between low-risk and high-risk. Between low-risk and high-risk, high-risk resulted in lesser values in kinetics (i.e., BRFD: low-risk = 4,518 ± 1,725 N/s, high-risk = 2,019 ± 761 N/s and PNI: low-risk: 238 ± 33 N.s, high-risk: 180 ± 35 N.s) and greater values in kinematics measures (i.e., KF: low-risk = 102.6 ± 10.4°, high-risk = 114.3 ± 13.0°, and BP: low-risk = 0.23 ± 0.05 s, high-risk = 0.32 ± 0.07 s), although BNI was significantly greater in the moderate-risk cluster among clusters (low-risk: 111 ± 21 N.s, moderate-risk: 122 ± 18 N.s, high-risk: 88 ± 16 N.s). Representation of MOCs movement strategy from each cluster are presented in Figure 5.

**TABLE 6:** Markerless motion capture and force plate variables used for K-means clustering in Marine Officer Candidates.

	Abbreviation	C1 (low-risk)	C2 (moderate-risk)	C3 (high-risk)	Omnibus *p* value	Bonferroni adjusted post hoc pairwise comparison *p*-value		
						C3,C1	C2,C1	C3,C2
**Kinetic measures**								
Braking RFD (N/s)	BRFD	4,518 ± 1,725	4,200 ± 1,556	2,019 ± 761	<0.001	<0.001	0.057	<0.001
Braking net impulse (N.s)	BNI	111 ± 21	122 ± 18	88 ± 16	<0.001	<0.001	<0.001	<0.001
Propulsive net impulse (N.s)	PNI	238 ± 33	228 ± 30	180 ± 35	<0.001	<0.001	0.004	<0.001
Peak relative propulsive power (W/kg)	PRPP	59 ± 7	51 ± 6	46 ± 6	<0.001	<0.001	<0.001	<0.001
**Kinematic measures**								
Braking phase (s)	BP	0.23 ± 0.05	0.27 ± 0.05	0.32 ± 0.07	<0.001	<0.001	<0.001	<0.001
Propulsive phase (s)	PP	0.33 ± 0.04	0.40 ± 0.04	0.42 ± 0.05	<0.001	<0.001	<0.001	<0.001
Max Hip Flexion (degrees)^a^	HF	89.8 ± 15.9	104.3 ± 13.3	102.6 ± 15.6	<0.001	<0.001	<0.001	0.712
Max Knee Flexion (degrees)^a^	KF	102.6 ± 10.4	126.0 ± 9.4	114.3 ± 13.0	<0.001	<0.001	<0.001	<0.001
Max Ankle Flexion (degrees)^a^	AF	29.6 ± 4.9	36.8 ± 5.3	33.0 ± 5.6	<0.001	<0.001	<0.001	<0.001
Dynamic Valgus (degrees)^a^	DV	5.4 ± 2.8	5.5 ± 3.2	6.4 ± 3.4	<0.001	0.002	1.0	0.007

a mMoCap variables; One-way ANOVA, analyze effect of cluster (between-subjects variable: C1, C2, C3); Figure 5A (Left). = low-risk cluster, Figure 5B (Middle). = moderate-risk cluster, Figure 5C (Right). = high-risk cluster; data presented as: mean ± standard deviation.

## Discussion

This study applied unsupervised learning (k-means clustering) to baseline CMJ kinetic and kinematic variables to discriminate distinct movement strategies that were prospectively associated with developing an MSKI during OCS in Marine Officer Candidates. Significant differences in the variables used to designate each cluster were indicative of greater kinetics (BRFD, impulse, power) and lower/shorter kinematics (joint flexions, time duration measures) in the low-risk cluster, while the high-risk cluster demonstrated longer time durations and moderate joint flexion kinematics, and lower kinetics. Such findings suggest that efficient CMJ movement strategies are associated with a lower risk for MSKI during military training.

### K-Means Clustering has Utility for Classifying MSKI Risk

Two separate analyses were conducted in this study to evaluate MSKI risk in Marine Officer Candidates. When utilizing a two-way ANOVA to compare between sexes and injury groups, kinetic variables, such as BRFD, braking net impulse, and propulsive net impulse were significantly greater in the noMSKI group compared to MSKI. No kinematic variables were significantly different between MSKI and noMSKI, suggesting that CMJ kinematics are not associated with MSKI risk. In agreement with our results, similar analyses were previously reported using multiple independent sample t-tests to evaluate running gait kinematics and reported few kinetics and kinematics were significantly different between MSKI and noMSKI in runners (Dudley et al., 2017). Additionally, some kinetic and kinematic measures in the single-leg drop jump were significantly different between male and female adolescents (Romanchuk et al., 2020). The interpretation from these analyses are limited, because each individual kinetic or kinematic variable are compared across groups (MSKI vs. noMSKI or Male vs. Female) one at a time, whereas in reality, the kinematic chain functions as a synchronized movement of multiple components. Therefore, clustering movement strategies incorporates combinations of kinetics and kinematics that relate to MSKI risk and sex differences. Such approaches allow not only for clustering of CMJ variables to create a comprehensive movement profile, but also compare MSKI status and sex between movement strategies rather than grouping based on MSKI classification.

When comparing kinetic and kinematic variables among injury groups, there are often substantial overlap of variables, which limits significant results between groups that contribute to MSKI risk associations (Bahr, 2016). This highlights the need for analyses to distinctly stratify groups (i.e., clustering) and then observe MSKI and sex proportions for clear thresholds of variables associated with varying MSKI risk with minimal overlap of variables. Recently, Rauch et al. (2020) used k-means clustering to stratify three groups to evaluate the CMJ kinetic and kinematic variables associated with basketball player positional group movement strategies, concluding that CMJ movement strategies indicated positional groups (i.e., forward, guards). Our analysis followed a similar method and approach to understand CMJ movement strategies and their relation to MSKI. While k-means clustering is a readily used technique to stratify biomechanics data (i.e., gait pattern) it has rarely been used to classify proportions of MSKIs. Typical machine learning biomechanical analyses use predictive modeling [classification (80.6%) and regression (11.6%)] with limited use of data mining techniques, such as clustering (7.8%) (Halilaj et al., 2018). Due to differences in methodologies, it is difficult to compare the present results with previous literature, therefore this analysis highlights the utility of clustering techniques to identify MSKI risk associations.

In our clustering analysis, the proportion of MSKIs across movement strategy clusters significantly differed, in which the low-risk cluster had the lowest proportion of MOCs with a MSKI (13.8%), followed by moderate-risk (22.5%) and high-risk (30.5%). Similar associations were present for the proportion of MSKIs in male and females MOCs, in which the highest proportion of MSKI were in the high-risk cluster. Although, female MOC's proportions of MSKIs across clusters were not significantly different, potentially due to a smaller sample size than male MOCs. In addition, opposite trends existed where female MOCs were highly distributed to the high-risk cluster and male MOCs in the low and moderate-risk cluster. Therefore, we have demonstrated that female MOCs have different CMJ movement strategies than male MOCs that associate with a higher risk of MSKI across clusters. However, there was a large proportion of male MOCs (59%) with similar CMJ movement strategies as female MOCs demonstrating that movement strategies, along with female sex are risk factors for higher MSKI risk.

### Kinetic and Kinematic Variables Associate With the MSKI Risk Clusters

The MSKI risk labeled proportions (high, moderate and low risk) coincided with kinetic values, such that higher values associated with low-risk (except for breaking net impulse), and lower values associated with high-risk (see Table 6). On the other hand, the low-risk cluster had lower/shorter kinematics (joint flexion, time duration), while the moderate and high-risk cluster had higher/longer kinematic values. The trends of variables across groups parallel similarly to those reported by McHugh et al. (2021). Specifically, when distributing male NCAA athletes across three groups (average, below average, and above average) FP variables, inverse trends were observed. Peak relative propulsive power was highest (63.8 ± 3.2 W/kg) and propulsive phase was lowest (0.268 ± 0.025 s) in the above average group, while peak relative propulsive power was lowest (52.3 ± 1.9 W/kg), and propulsive phase was highest (0.295 ± 0.041 s) in the below average group. In addition, Márquez et al. (2018) reported that recreationally trained athletes’ relative peak power was greater in men (52.91 ± 7.13 W/kg) than women (43.36 ± 2.58 W/kg). These values suggest that the MOCs in the moderate-risk cluster, predominantly male MOCs (96.2%), were more similar to both the below average male NCAA and male recreationally trained athletes. While the high-risk cluster MOCs (41% female and 59% male MOCs) were lower in peak relative propulsive power when compared to below average male NCAA and male recreationally trained athletes, but greater than female recreationally trained athletes. As the military emphasizes enhanced training and preparation to optimize performance for the “tactical athlete,” it may be advantageous for MOCs to meet similar thresholds of performance that have similar training demands as an average NCAA D1 athlete. In addition, as the military facilitates gender integration (male and female service members in the same roles, i.e., ground combat), the baseline strength and power gap between sexes should be attenuated, as the training demands for male and female MOCs are similar at OCS.

As previously mentioned, there is limited research in clustering with MSKI risk association. There is also limited research regarding the utility of the CMJ for classifying MSKI risk. More prevalent is the drop landing and drop jump for risk indicators of MSKI, specifically anterior cruciate ligament, and patellofemoral pain (Krosshaug et al., 2016; Holden et al., 2017; Boling et al., 2021). During the drop landing, females are at risk for greater knee valgus at initial contact (Lam and McLeod, 2014; Prieske et al., 2015). In our sample, dynamic valgus was significantly greater in the high-risk cluster (6.4 ± 3.4°) comprising 41% of the female MOCs, when compared to the low-risk cluster (5.4 ± 2.8°), which could be postulated to be a risk factor for greater MSKI risk, although a 1-degree difference may not be clinically relevant.

Due to the lesser joint flexions in the low-risk clusters for hip (89.8 ± 15.9°), knee (102.6 ± 10.4°), and ankle (29.6 ± 4.9°), there were shorter time durations (i.e., braking phase), but greater kinetics (i.e., BRFD) thus indicating these MOCs were more efficient in the CMJ movement strategies, with greater energy storage utilization, such as the stretch shortening cycle (Komi, 2000) in the amortization phase (McMahon et al., 2018). Alternatively, the high-risk and moderate-risk cluster had greater degrees of flexion in all joints with longer time durations for the braking and propulsive phase. The moderate-risk cluster had approximately two times the BRFD values and significantly greater braking net impulse, propulsive net impulse measures than the high-risk cluster, concluding that both moderate-risk and high-risk clusters were less efficient in CMJ variables due to their underlying kinematics, but the moderate-risk cluster was protected by greater kinetic measures (peak relative propulsive power, braking net impulse, propulsive net impulse and BRFD) than the high-risk cluster.

### Joint MSKI Location is Associated With Movement Strategies

Hip, Knee and Ankle MSKI proportions were calculated in each cluster (Table 5), with the proportion of MOCs with an ankle MSKI (Table 5A) significant across clusters, but not hip or knee MSKI. Ankle sprains have been attributed to poor transition of non-weight bearing to weight bearing conditions during dynamic movements (Delahunt and Remus, 2019). In the high-risk cluster, there is a low braking impulse and rate of force development, thus pre-disposing these individuals if repetitive loading occurs, causing fatigue in the braking phase or a scenario if the external force exceeds their capacity to develop force. Lastly, hip extension strength was reported to be a significant indicator of an ankle sprain (De Ridder et al., 2017). While not directly measured, the CMJ relies heavily on the musculature around the hip (Mackala et al., 2013) and may contribute to the significant findings. Interestingly, the proportion of knee MSKI did not follow the same distribution as ankle and hip MSKI, with the largest proportion of knee MSKIs in the moderate-risk cluster (Table 5B). This could be related to greater knee flexions, lower performance in the propulsive phase, but similar BRFD when compared to the low-risk cluster. This demonstrates the moderate-risk cluster lacked the strength to transition and apply force into the propulsive phase due to the large degrees of flexion. This is turn, over acute repetitive dynamic loading and take-off, may lead to fatigue in the musculature causing structural properties around the knee joint to be at a greater risk for MSKI (Marshall et al., 2014). In addition, patellofemoral stresses are greatest at the deepest degrees of knee flexion, thus contributing to the potential of chronic overuse MSKIs (Schoenfeld, 2010).

### The Countermovement Jump, a Useful Tool for MSKI Risk Screening

The high-risk cluster had a relative risk (RR) 2.2 times higher risk for developing an MSKI compared to the low-risk cluster when grouping dependent variables in the CMJ for both male and female MOCs. In a recent systematic review, Pedley et al. (2020), reported that 11/14 = 78.6% of articles reviewed reported MSKI associations with drop-jump, while only 1/12 = 8.3% articles reviewed reported MSKI associations with CMJ. Interestingly, many of these articles in the review by Pedley et al. solely evaluated CMJ jump height. Knapik et al. (2001) reported no risk associations with MSKI when vertical jump height was stratified by higher jump heights vs. lower heights in men and women during U.S. Army Basic Combat Training. While jump height is a readily utilized measure to identify neuromuscular readiness and MSKI risk, the current state of force plate technology enables practitioners to readily identify different phase characteristics (braking and propulsive phase) as simply as calculating jump height (Lake et al., 2018). In addition, Knapik et al. (2001) reported Army Physical Fitness Test measures and reported that slower 3.2-km run time [RR (CI) = Men: 1.6 (1.0–2.4) and Women: 1.9 (1.1–2.5)] and fewer push-ups [RR (CI) = Men: 1.8 (1.2–2.8) and Women: 1.6 (1.1–2.5)] were significant contributors to MSKI risk associations when compared to faster 3.2-km run times and greater push-ups. This further demonstrates that CMJ kinetic and kinematic variables, when stratifying by high and low performers, have similar utility in classifying MSKI risk associations as physical fitness test in the military.

### Limitations and Strengths

Strengths of this study include MSKI diagnosing and reporting by the same medical staff through all OCS classes. Since OCS has the same requirements for all MOC, the training is similar regarding volume, load, and duration, and allowed for a controlled environment mitigating confounders in physical training. Limitations of this study were the relatively smaller sample sizes when MOCs were stratified by MSKI anatomic sub-location and then further by sex. In addition, because a MSKI may result in attrition from OCS or a light duty classification, it is possible that some of the MOCs with noMSKI did not seek medical attention. While we have suggested thresholds with variables according to MSKI risk (Table 6), these thresholds may not be generalizable to other populations (i.e., NCAA athletes). As force plates begin to become mainstream in usage for tactical and athletic populations, we encourage researchers to report multiple descriptive measures (Tables 2, 6) for carryover of population similarity. We have demonstrated the CMJ variables indicate MSKI using multiple domains of technology (i.e., FP and mMoCap), although novel, backend cleaning of data and filtering of artifacts consumed time in the final interpretation of results. In a dynamic military setting needing quick actionable decision aids, future software updates or technology should include automatic re-processing of data or flags in artifacts of data. In addition, the proprietary MSKI risk algorithms used by the commercial grade technology, should be properly validated before use in a population.

## Conclusion

This study demonstrates that CMJ movement strategies are associated with MSKI risk in military populations. By utilizing robust analytical techniques (i.e., unsupervised cluster analysis), we successfully identified three distinct CMJ movement strategies that differed in the proportion of MOCs with a MSKI, such that the high-risk cluster had a relative risk of developing a MSKI 1.6 and 2.2 times higher than the moderate and low risk clusters, respectively. These data further provide thresholds for practitioner use, to make actionable decisions for interventions to modify CMJ strategies (i.e., joint flexions) and/or implement auxiliary training programs to improve strength and power, and thus, reduce MSKI. As the field of human performance expands into military populations, combined with the growing market of technology used for screening, it is necessary to understand the applicability and usefulness of screening tools in this population.

## Data Availability

The datasets presented in this article are not readily available because of the contracting through the Office of Naval Research. Requests to access the datasets should be directed to Dr. Bradley Nindl, bnindl@pitt.edu.

## AUTHORS NOTE

This work was funded by ONR N00014-20-C2020, the views expressed in this paper are those of the authors and do not reflect the official policy of the Department of Army/Navy/Air Force, Department of Defense, or the U.S. Government.
